# *MEDIATOR18* and *MEDIATOR20* confer susceptibility to *Fusarium oxysporum* in *Arabidopsis thaliana*

**DOI:** 10.1371/journal.pone.0176022

**Published:** 2017-04-25

**Authors:** Thorya Fallath, Brendan N. Kidd, Jiri Stiller, Celine Davoine, Stefan Björklund, John M. Manners, Kemal Kazan, Peer M. Schenk

**Affiliations:** 1 Plant-Microbe Interactions Laboratory, School of Agriculture and Food Sciences, The University of Queensland, St Lucia, Australia; 2 CSIRO Agriculture and Food, Queensland Bioscience Precinct, St Lucia, Australia; 3 Department of Medical Biochemistry and Biophysics, Umeå Plant Science Center, Umeå University Umeå Sweden; 4 CSIRO Agriculture and Food, Black Mountain, Canberra, Australia; 5 Queensland Alliance for Agriculture & Food Innovation (QAAFI), University of Queensland, St Lucia, Australia; National Taiwan University, TAIWAN

## Abstract

The conserved protein complex known as Mediator conveys transcriptional signals by acting as an intermediary between transcription factors and RNA polymerase II. As a result, Mediator subunits play multiple roles in regulating developmental as well as abiotic and biotic stress pathways. In this report we identify the head domain subunits MEDIATOR18 and MEDIATOR20 as important susceptibility factors for *Fusarium oxysporum* infection in *Arabidopsis thaliana*. Mutants of *MED18* and *MED20* display down-regulation of genes associated with jasmonate signaling and biosynthesis while up-regulation of salicylic acid associated pathogenesis related genes and reactive oxygen producing and scavenging genes. We propose that MED18 and MED20 form a sub-domain within Mediator that controls the balance of salicylic acid and jasmonate associated defense pathways.

## Introduction

Being sessile in nature, plants require finely tuned response pathways to adapt to the environment around them. Facing challenges from both abiotic and biotic stresses, plants require sensors to perceive external signals and a signaling pathway that leads to transcriptional activation through DNA-binding transcription factors (TFs). Control over which pathway in the broader plant signaling network is activated is crucial to produce the correct response and to prevent misallocation in energy production [1, 2]. One example of a finely-tuned network can be seen in plant defense signaling pathways, where successful recognition of a pathogen modulates the output of defense genes that are transcribed. For instance, it is generally accepted that the model plant *Arabidopsis thaliana* will activate genes from the salicylic acid (SA) associated pathway in response to infection by biotrophic pathogens whereas genes associated with the jasmonate (JA) and ethylene (ET) associated pathways are activated strongly in response to necrotrophic pathogens [3]. While defense pathways are likely to be more complex when considered as a network [4, 5], antagonistic regulation of the SA and JA/ET defense pathways allow prioritization of the defense response for maximum effectiveness against the pathogen that is invading [6].

To overcome plant defenses, plant pathogens may modify hormone signaling to create an environment suitable for colonization [7]. For instance, the bacterial pathogen *Pseudomonas syringae* pv. *tomato* (*Pst*) is known to affect the abscisic acid, auxin, JA, and SA signaling pathways [8–10]. *Pst* uses the JA-isoleucine (JA-Ile) hormone mimic coronatine to activate the JA associated transcription factor MYC2, which then activates the NAC TF genes, *ANAC019*, *ANAC055* and *ANAC072* to suppress SA biosynthesis and metabolism [11]. Similarly, isolates of the hemi-biotrophic fungal pathogen, *Fusarium oxysporum* have been shown to produce JA-Ile and other JA conjugates to help infect *A*. *thaliana* [12, 13]. Mutants in the JA-Ile receptor CORONATINE INSENSITIVE1 (COI1) show strong resistance to *F*. *oxysporum* isolates that produce JA-Ile in culture, while the *myc2* mutant and an activation allele of the JAZ7 transcriptional repressor have enhanced resistance and susceptibility, respectively [13–17]. The resistance phenotype of *coi1* was shown to be independent of SA-associated defense genes using the *NahG* transgene [15], however exogenous application of SA to the leaves was found to increase resistance [18]. *F*. *oxysporum* has been shown to induce JA-associated gene expression as well as tryptophan secondary metabolism during infection [19, 20]. Activation of the tryptophan metabolic pathway leads to increased auxin production. Interestingly, auxin signaling and transport genes, but not auxin biosynthetic genes have been shown to be involved in susceptibility to *F*. *oxysporum* [20]. Lastly, the ethylene receptor mutant *etr1-1* and the abscisic acid (ABA) biosynthetic mutant *aba2-1* show increased resistance to *F*. *oxysporum* suggesting ethylene and ABA are also required for susceptibility [14, 21].

With a complex transcriptional network to co-ordinate, plants have evolved a large number of TFs to fine tune gene expression. For instance, the *A*. *thaliana* genome contains over 1500 transcription factors [22, 23]. To process information from a large number of TFs, eukaryotes possess a protein complex called Mediator that relays the signal from TFs to RNA Polymerase II. Not surprisingly, mutations in the Mediator complex have been found to affect a wide range of plant developmental processes as well as abiotic and biotic stress responses [24, 25].

In a screen of 12 individual *A*. *thaliana* Mediator subunits, we previously identified the *med25* and *med8* mutants to be moderately resistant to *F*. *oxysporum* [26]. The *med25* mutant was found to be partially insensitive to JA and showed a reduction in JA-associated gene expression [26, 27]. As insensitivity to JA has been linked to *F*. *oxysporum* resistance [15], the *med25* mutant was also hypothesized to be resistant due to an attenuation of JA signaling. In contrast, the *med8* mutant had no change in JA-associated defense gene expression and a *med25 med8* double mutant led to additive resistance over the single mutations [26]. This suggested a potentially separate mechanism for susceptibility to *F*. *oxysporum* that is conferred by MED8. The MED8 subunit is predicted to be located in the Head domain of the Mediator complex and is thought to act as a linker with the “movable jaw” subunits MED18 and MED20 based on structural conservation between eukaryotic Mediator complexes [28, 29].

In this study, we examined mutants of the *A*. *thaliana MED18* and *MED20* genes and found them to be highly resistant to *F*. *oxysporum*. JA signaling genes were significantly reduced under *F*. *oxysporum* infection in *med18* and *med20*, while expression of the SA-associated genes, *PATHOGENESIS RELATED1 (PR1)* and *PATHOGENESIS RELATED5* (*PR5*) as well as several genes associated with reactive oxygen production were up-regulated. Our data suggest that the MED18-MED20 sub-module of the Mediator complex confers susceptibility to *F*. *oxysporum* and modulates crosstalk between JA- and SA-associated defense pathways.

## Materials and methods

### Plant growth conditions

*A*. *thaliana* Col-0, *med18* and *med20* seeds were sown into autoclaved University of California mix soil and kept at 4°C in the dark for 48 hours. After stratification, plants were grown at 24°C, with an 8 hour photoperiod (160 μE m^-2^s^-1^) and 60% humidity, with a night time temperature of 21°C and humidity of 70%. After 2 weeks, seedlings were gently removed from the soil and transferred to 30-well trays, and grown until the six to eight leaf stage until inoculation with *F*. *oxysporum* or treatment with hormones. SA treatment was performed according to [30]. Grafting of WT and *med18* plants was performed as described [15] using young seedlings grown on ½ MS agar in long day conditions (16 hour photoperiod (160 μE m^-2^s^-1^) and 60% humidity, with a night time temperature of 21°C and humidity of 70%). Once the grafts had formed, plants were gently transferred to soil for three weeks before inoculation with *F*. *oxysporum*. Flowering time assays were also conducted under the same long day conditions, and the number of rosette leaves recorded at the initiation of flowering. The *med18* seeds (salk_027178C) and *med18-1* (sail_889_C08) were obtained from the Arabidopsis Biological Resource Center while the *med18-1;MED18-HA* seeds were a kind gift from Tesfaye Mengiste. The *med20* seeds contain a C-to-T mutation in At2g28230 (MED20a) resulting in a premature stop, and were a kind gift from Xuemei Chen [31].

### *F*. *oxysporum* inoculation

Plants were inoculated with *F*. *oxysporum* isolate 5176 as described previously [26]. Briefly, at 1 h after the start of the photoperiod, plants were gently uprooted and dipped for fifteen seconds in a spore suspension with a concentration of 1 x 10^6^ spores/mL in water and then replanted. Mock plants were dipped in water and replanted.

### *F*. *oxysporum* colonization scoring

The *β-GLUCURONIDASE* (*GUS*) expressing *F*. *oxysporum 5176* strain was a kind gift from U. Schumann [32]. GUS staining was performed according to [33]. Analysis of GUS staining was performed on ten plants of both WT and *med18*, which were gently uprooted twelve days after infection and washed in distilled water before being vacuum infiltrated in staining solution and incubated at 37°C. GUS stained roots were imaged under a compound microscope (Nikon) and scored for the presence of GUS as a percentage of the total root length using ImageJ.

### Yeast-2-hybrid screening

To test the interaction between plant Mediator subunits, the full length coding sequence of MED8, MED18, MED20 and MED21 were amplified with primers listed in S1 Table from *Arabidopsis* Col-0 cDNA. The PCR products were cloned into the vector pCR8GW-TOPO according to the manufacturer’s instructions (Invitrogen). All plasmid constructs were confirmed by restriction analysis and sequencing. The GAL4-DNA binding domain bait constructs (DBD-MEDs) were generated by an LR recombination reaction with pCR8GW–MEDs and pDEST32. The GAL4 Activation domain prey constructs (AD-MEDs) were generated by LR recombination reaction with pDEST22 and the pCR8GW–MED plasmids. The LR recombination reaction was performed using the Gateway LR Clonase II Enzyme kit (Invitrogen) according to the manufacturer’s instructions and checked using restriction digests. The pDEST32-GFP (DBD-GFP) control vector was a kind gift from Volkan Cevik. Yeast-2-hybrid interactions were performed by transforming bait and prey plasmids into the yeast strain *S*. *cerevisiae* (MaV203). The transformed cells were plated onto synthetic complete medium minus leucine and tryptophan SC-Leu-Trp (-LW) plates before streaking onto fresh plates. Freshly streaked cultures were then resuspended in 50ul sterile water and serially diluted ten times and plated on SC-Leu-Trp (-LW) and SC-Leu-Trp-His (-LWH) plates (Sunrise Science) containing 12.5mM 3-aminotriazole (3-AT) (Sigma). The Yeast-2-Hybrid interaction experiment was repeated with separate transformations and showed the same results.

### RNA sequencing

Infected root tissues of Col-0, *med18* and *med20* were harvested 24 h after inoculation with *F*. *oxysporum* (three independent biological replicates of 20 plants each). Total RNA was isolated using an RNeasy Plant Mini kit (Qiaqen) and RNA quality checked using a Nanodrop ND-1000 (Nanodrop) and an Agilent 2100 Bioanalyser (Agilent Biotechnologies). Library preparation and RNA sequencing on the infected root RNA samples were performed by the Australian Genome Research Facility (AGRF). Messenger RNA was selected using Poly-A tail selection prior to preparation of 100bp paired-end libraries. Sequencing was performed on an Illumina HiSeq 2000 system generating approximately 23 million raw RNAseq reads per sample. Fastq files are available at the NCBI Sequence Read Archive (SRA) under study number SRP092151. Differential expression analysis was performed using the Tuxedo analysis suite [34]. Briefly, Bowtie2 along with Tophat were used to align generated reads to the TAIR10 *A*. *thaliana* reference genome. After expressed transfrags were assembled, Cufflinks was used to quantify gene abundance and transcriptome assemblies were then merged using Cuffmerge. Statistical analysis was performed within the Cufflinks analysis with false discovery rate and correction for multiple comparisons applied using standard run parameters. Genes considered differentially expressed showed a statistically significant difference in expression values (P<0.05) and a Log2 fold change >1. Venn diagrams were produced using Venny 2.1 (http://bioinfogp.cnb.csic.es/tools/venny/index.html) and a heatmap produced using matrix2png [35].

### Real-time quantitative reverse transcriptase PCR (Real time qRT-PCR) analyses

Real time qRT-PCR expression analysis was performed on hormone treated leaves as well as mock or *F*. *oxysporum* infected roots. Leaf and root tissues were collected at 24 hours after treatment for gene expression studies. Salicylic acid (SA) was obtained from Sigma-Aldrich. SA treatment was performed by lightly and evenly spraying leaves with 100μM SA. Total RNA was isolated using an RNeasy Plant Mini kit (Qiaqen) and RNA quality checked using a Nanodrop ND-1000 (Nanodrop). cDNA synthesis was performed using SuperScriptIII (Thermo Fisher) and real time quantitative reverse transcriptase PCR was performed using an ABI ViiA7 Sequence Detection System (Applied Biosystems). Each reaction contained 5 μL of SYBR Green (Applied Biosystems) and 1 μL of 3 nM of each gene-specific primer pair and 4 μL of cDNA template to a final volume of 10 μL. The PCR primer efficiency (E) of each primer pair in each individual reaction was calculated from the changes in fluorescence values (ΔRn) of each amplification plot, using LinReg PCR software [36]. Amplification plots were analyzed using a threshold of 0.20 to give a cycle threshold (Ct) value for each gene and cDNA combination. Gene expression levels relative to the *Arabidopsis* housekeeping genes *β-ACTIN 2* (AT3G18780), *β-ACTIN 3* (AT3G53750) and *β-ACTIN 7* (AT1G49240) were calculated for each cDNA sample using the following equation: The gene transcript levels relative to actin = (E gene^(-Ct gene)) / (E *Actin* ^(-Ct *Actin*)). The primer pairs used in Real time RT-qPCR (S1 Table).

## Results

### *MED18* and *MED20* confer susceptibility to *F*. *oxysporum*

**Fig 1:**
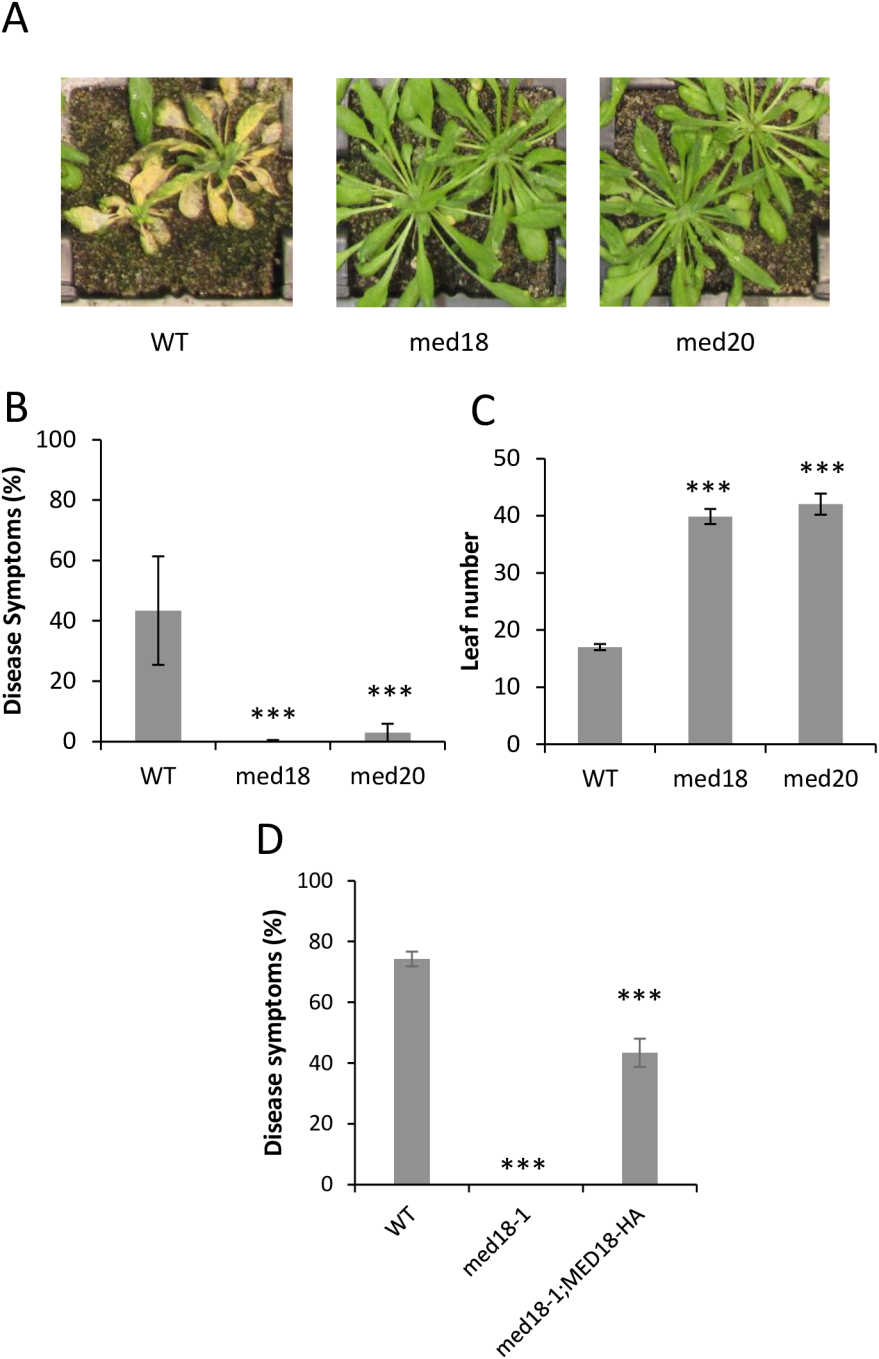
MED18 and MED20 are *F*. *oxysporum* susceptibility factors. (A) Typical disease symptoms of WT (Col-0), *med18* and *med20* plants 14 days after infection with *F*. *oxysporum*. (B) Average percentage of leaves showing chlorosis at 14 days after infection. Inoculations consisted of three biological replicates with each containing twenty plants. (C) Number of rosette leaves at flowering when grown under long-day conditions. Rosette leaves were counted at the first sighting of the floral bud at approximately 0.5cm in length. (D) Disease symptoms of *med18-1* and *med18-1;MED18-HA* plants 18 days after infection with *F*. *oxysporum*. *** represents significance (p<0.001) using Student’s T-test of each mutant compared to the WT. Error bars represent standard error.

The *Arabidopsis MED8* and *MED25* genes were previously found to confer susceptibility to *F*. *oxysporum* [26]. MED25 has been shown to interact with MED16 in *Arabidopsis* which is located in the Tail domain of Mediator [37], while MED8 is located in the Head domain. We investigated whether the *Arabidopsis* head domain subunits, MED18 and MED20, which have been shown to be located adjacent to MED8 in the yeast Mediator complex [28, 29], also conferred *F*. *oxysporum* susceptibility. We inoculated *med18* (salk_027178C) and *med20* [31] mutants with *F*. *oxysporum* and found them to be highly resistant with less than 3% of leaves showing disease symptoms compared to approximately 40% of leaves in the wild-type *A*. *thaliana* Col-0 (Fig 1A and 1B). As previously reported [31, 38], we found that *med18* and *med20* plants had a strong delay in flowering time and showed a similar phenotype to the *med8* mutant with approximately forty leaves being produced before flowering in long day (LD) conditions (Fig 1C; [26]). Although there are no T-DNA insertion mutants available for *med20*, we were able to screen a second *med18* insertion mutant (previously referred to as *med18-1* [39]) and a complemented *med18-1* line expressing a hemagglutinin (HA)-tagged MED18 construct [39]. The *med18-1* line showed complete resistance to *F*. *oxysporum* while the complemented line showed a partial restoration of susceptibility, suggesting the HA-Tag may potentially affect MED18’s role in mediating *F*. *oxysporum* susceptibility (Fig 1D).

### MED18 interacts with MED20 in Y2H experiments

**Fig 2:**
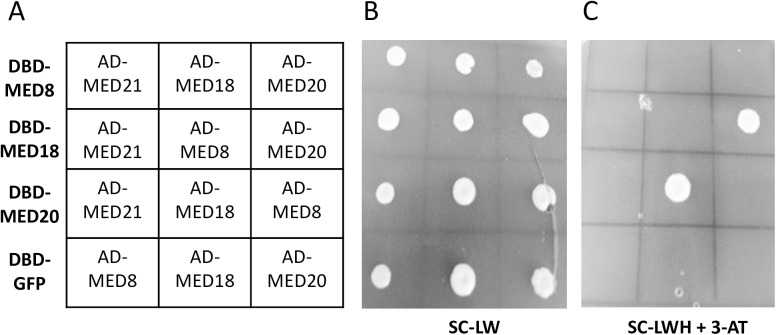
MED18 and MED20 interact in Yeast-2-Hybrid experiments. GAL4 DNA-Binding Domain (DBD-) plasmids and GAL4 Activation Domain (AD-) plasmids were co-transformed into *S*. *cerevisiae* and plated onto synthetic complete medium lacking Leucine and Tryptophan (SC-LW) or synthetic complete medium lacking Leucine, Tryptophan and Histidine but containing 12.5mM 3-aminotriazole (SC-LWH +3-AT). The plate layout is shown in (A) where each row contains the same DBD-MED or DBD-GFP plasmid with the co-transformed AD-MED plasmid shown in the 3x4 grid. (B) The transformants plated on SC-LW. (C) The transformants plated on SC-LWH +3-AT showing reciprocal positive interactions between MED18 and MED20 plasmids. The experiment was repeated with independent transformations and showed the same result.

As, *med8*, *med18* and *med20* mutants have similar pathogen and developmental phenotypes, we examined whether MED8, MED18 and MED20 interact in Yeast-2-Hybrid assays. As negative controls we included GFP and MED21 which are not expected to interact with MED18 or MED20 based on the yeast Mediator architecture. We found a positive interaction between MED18 and MED20 in reciprocal GAL4-Activation Domain and GAL4-DNA-Binding Domain fusions, however we did not detect positive interactions with MED8, MED21 or GFP (Fig 2). While more research is required on the structure of the *Arabidopsis* Mediator complex, these results suggest that MED18/MED20 may form a subdomain of the Mediator head complex that controls both flowering time and susceptibility to *F*. *oxysporum* in *Arabidopsis*. Additional experiments are required to demonstrate a direct interaction between MED18 and MED20 *in vivo*.

### *med18* roots are required for *F*. *oxysporum* resistance but not the late flowering phenotype

As loss of either of MED18 or MED20 leads to similar defense and flowering phenotypes in *A*. *thaliana*, we chose to focus on MED18 to further investigate how MED18 and MED20 affect resistance to *F*. *oxysporum*. Prior to the identification of the *med18* and *med20 F*. *oxysporum* resistance phenotypes, the only mutant identified to have comparable reductions in disease symptoms was a mutant in the JA-Ile receptor *COI1*, [13, 15, 16]. Through grafting experiments, we previously showed that the *coi1* rootstock was responsible for resistance as grafts made with the *coi1* rootstock showed a strong reduction in leaf symptoms regardless of the shoot genotype [15]. Thus, we performed grafting experiments with the *med18* mutant to determine whether resistance is also determined by the roots.

**Fig 3:**
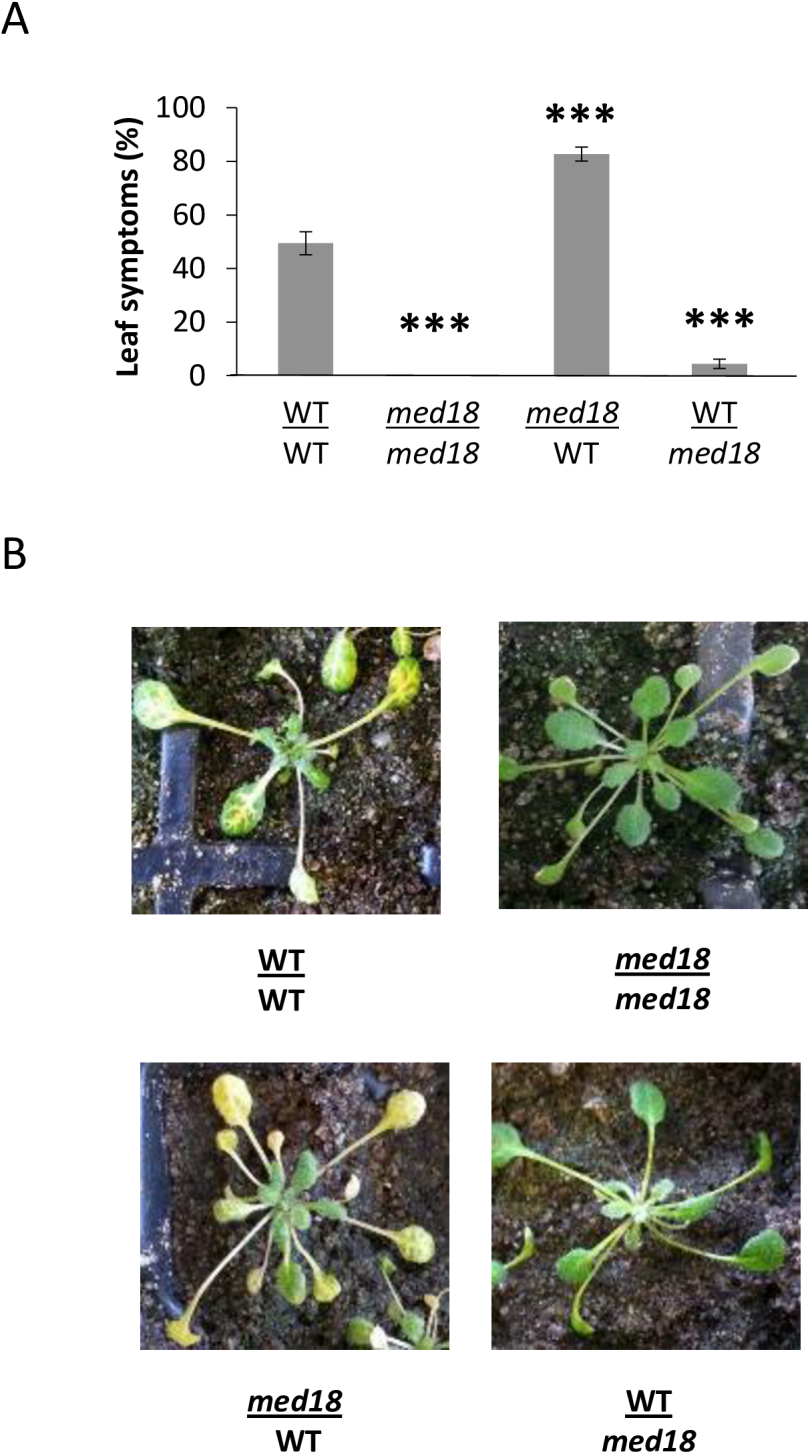
The resistance of *med18* is mediated by the roots. Reciprocal grafts made between the WT (Col-0) and *med18* seedlings revealed that roots containing the *med18* root genotype were highly resistant to infection, whereas *med18/*WT or WT/WT self-grafts were highly susceptible. (A) shows the average percentage of leaf chlorosis from eighteen plants per graft combination. Error bars represent standard error. (B) shows typical disease symptoms at 12 days after infection. *** represents significance (p<0.001) using Student’s T-test of each mutant compared to the WT/WT self-graft.

Prior to grafting, the seedlings were grown in long day conditions and therefore have an accelerated flowering time. The delay in flowering time in *med18* was retained in the grafting process with grafts containing the WT Col-0 scion transitioning to flowering whereas the grafts with *med18* scions remained vegetative. As a delay in flowering time had previously been associated with increased *F*. *oxysporum* resistance [26, 40] we examined whether the transition to flowering in the grafted plants affected resistance to *F*. *oxysporum*. Interestingly, despite undergoing flowering during the time of inoculation, the *med18* rootstock: Col-0 scion graft was resistant to *F*. *oxysporum* infection, whereas the Col-0 rootstock: *med18* scion was susceptible (Fig 3 and S1 Fig). These results show that, similar to *coi1*, the resistance of *med18* to *F*. *oxysporum* is dependent on the root genotype. In addition, delayed flowering time and *F*. *oxysporum* resistance are spatially independent traits in the *med18* mutant.

### *F*. *oxysporum* colonization is restricted in *med18* roots

**Fig 4:**
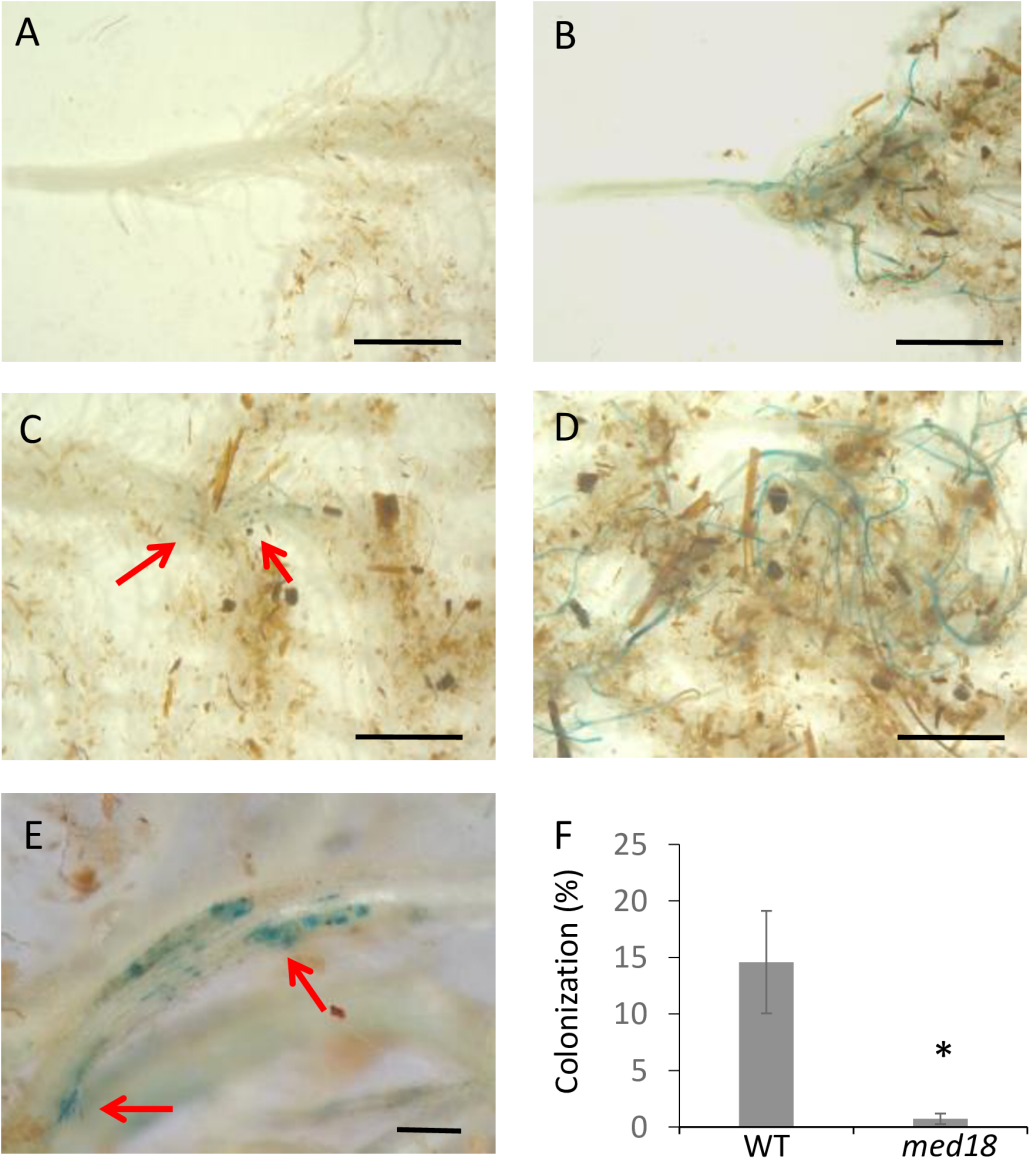
*F*. *oxysporum* shows restricted colonization in *med18* roots. Ten plants of each genotype were isolated twelve days post infection with a GUS-expressing strain of *F*. *oxysporum* and stained for GUS expression. Colonization of the *med18* (A, C, E) and WT (B, D) root system. Infection in the *med18* root system was localized to a small region in the roots and evidence of unsuccessful infection attempts were observed (red arrows). Photos shown in (C) and (D) are the lower portion of the root system seen in (A) and (B) respectively. Photos are representative of the results for each genotype. (A-D) are imaged using a Zeiss stereomicroscope at 4x zoom and (E) imaged using a Nikon compound microscope. Scale bars represent 5mm (A-D) and 100μm (E). Colonization was scored as a percentage of the total root mass and the average shown in (F). Error bars are standard error of ten individual plants. * represents significance using Student’s T-test (p<0.05).

As *med18* roots are responsible for resistance to *F*. *oxysporum*, we determined whether there is a reduction in root colonization in *med18* roots due to enhanced defenses or whether *med18* roots are similarly colonized but the pathogen is not able to cause disease in the shoots. We examined root colonization of *med18* with the use of a transgenic *F*. *oxysporum* constitutively expressing the *β-GLUCURONIDASE* (*GUS*) transgene [32]. Four week old WT and *med18* plants were root dipped in *GUS*-expressing *F*. *oxysporum* and the colonization of the root tissue was examined twelve days post infection. WT (Col-0) plants start to develop leaf symptoms around seven days post infection and at twelve days post infection the majority of WT plants were showing leaf symptoms and approaching collapse. In contrast the *med18* mutant plants fail to develop leaf symptoms and eventually proceed to flowering. A clear difference in root colonization was observed with WT plants showing extensive fungal colonization of the root system, whereas *med18* plants showed very limited colonization, with less than 5% of the roots positively stained for *GUS* (Fig 4A–4F). It was observed that WT plants showing chlorosis symptoms in the leaves often possessed strong colonization of a sub-section of the root, suggesting that the entire root system does not need to be colonized. This would explain why WT plants on average had only 15% of the root system colonized. However the colonization in these regions was extensive with the GUS expression in the WT roots appearing to spread along the root vascular tissue and connect through to adjacent lateral roots and up towards the hypocotyl (Fig 4B and 4D) similar to previous reports [41] whereas colonization in the *med18* line appeared to be punctate and rarely spread to adjacent lateral roots (Fig 4C and 4E). Therefore reduced leaf symptom development in the *med18* mutant correlates with restricted fungal colonization relative to WT roots.

### RNA sequencing analysis of *med18* and *med20* reveal a common gene regulon

**Fig 5:**
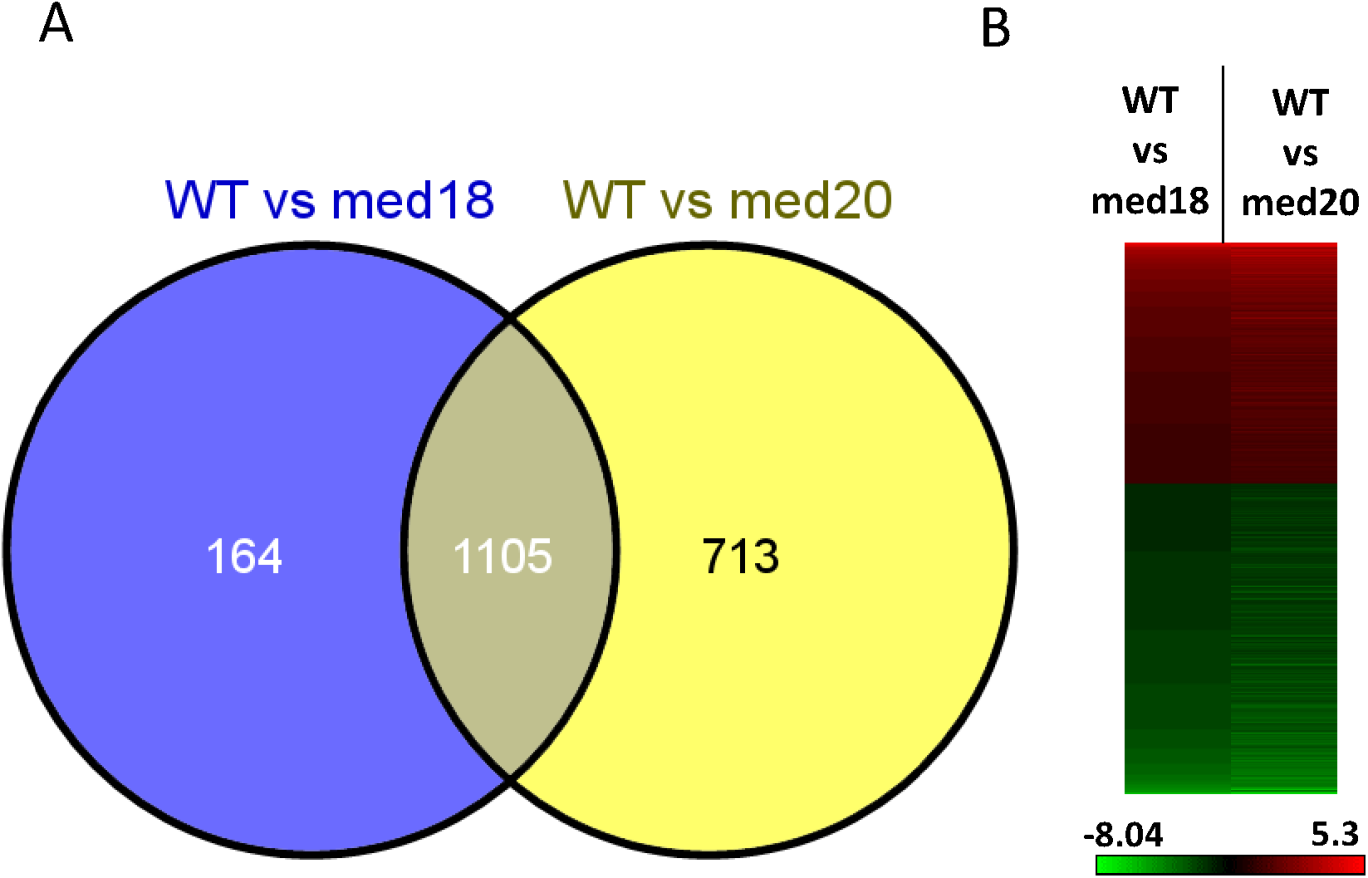
*MED18* and *MED20* co-regulate a similar subset of genes. (A) Venn diagram of the genes differentially expressed between WT and *med18* or WT and *med20*. (B) Heat map of the 1105 co-regulated genes. Heat map displays the Log2 fold change of *med18* or *med20* compared to the WT, with red representing higher and green representing lower expression in the Mediator mutants, respectively. Scale bar shows the colour change according to the Log2 fold change.

To identify genes that might be controlling resistance to *F*. *oxysporum* we conducted an RNA sequencing (RNAseq) experiment in WT, *med18* and *med20* roots infected with *F*. *oxysporum*. We chose to analyse early defense responses to *F*. *oxysporum* infection at one day post inoculation based on studies showing activation of defense gene expression in the roots at this time point [19]. Overall, 1269 and 1818 genes were differentially regulated (>2 fold and significant; FDR p<0.05) in *med18* and *med20*, respectively, compared to the WT after *F*. *oxysporum* infection (S2 Table). Comparing the WT vs *med18* and WT vs *med20* differentially expressed gene (DEG) lists to each other indicated a significant percentage of co-regulated genes with approximately 87% of *med18* DEGs and 61% of *med20* DEGs showing similar patterns of expression (Fig 5A and 5B). Thus the RNAseq analysis suggests that *MED18* and *MED20* regulate the expression of similar gene sets in *Arabidopsis*.

To investigate the genes that may be leading to enhanced resistance in the *med18* and *med20* mutants, we performed Gene Ontology (GO) enrichment analysis on the co-expressed genes [42]. The genes induced in the WT versus both *med18* and *med20* included several defence and stress related GO terms that were significantly enriched including: response to chitin, response to fungus, defense response, response to hormones (JA and abscisic acid) and plant cell wall modification (S3 Table). Examining the gene lists themselves, we found PAMP triggered immunity (PTI) associated genes such as *FLAGELLIN SENSITIVE 2* (*FLS2*), *PLANT U-BOX 23* (*PUB23*) and *MITOGEN-ACTIVATED PROTEIN KINASE 3* (*MPK3*) as being induced higher in the WT (Table 1). Also in the list were other genes associated with defence such as the cell wall synthases (*CESA4* and *CESA8*), the plant defensins *PDF1*.*4* and *PDF2*.*2*, as well as *PROPEP1*, which act as a precursor for the PEP1 peptide which activates defense genes and reactive oxygen species (ROS) [43]. In addition, several JA signaling genes were expressed higher in the WT relative to the mutants. These included the JA-associated transcription factor *MYC2*, the *JASMONATE ZIM DOMAIN* (*JAZ*) repressor proteins; *JAZ1*, *JAZ5*, *JAZ7*, *JAZ8* and *JAZ10*, JA biosynthesis genes *ALLENE OXIDE SYNTHASE* (*AOS*), *ALLENE OXIDE CYCLASE* (*AOC3*), *LIPOXYGENASE4* (*LOX4*), *SULFOTRANSFERASE 2A* (*ST2A*) which acts specifically on 11- and 12-hydroxyjasmonic acid [44], the galactolipase *DONGLE*, a JA-Ile-hydroxylase (*CYP94B3*) and *VEGETATIVE STORAGE PROTEINS*; *VSP1* and *VSP2* (Table 1). We confirmed the accuracy of the RNAseq analysis by examining the expression of *MYC2*, *PDF1*.*4*, *PDF2*.*2* as well as the differentially expressed *JAZ* genes using RT-qPCR (S2 Fig).

**Table 1:** The expression of plant defense and JA-associated genes is increased in the WT relative to *med18* and *med20* in response to *F*. *oxysporum* infection. Values represent the fold change in expression of the WT vs either *med18* or *med20* in the RNAseq experiment.

Category	Gene	Locus Identifier	WT vs *med18*	WT vs *med20*
Pattern triggered immunity	*FLAGELLIN-SENSITIVE 2 (FLS2)*	AT5G46330	3.22	3.65
	*PLANT U-BOX 23 (PUB23)*	AT2G35930	2.02	2.04
	*MITOGEN-ACTIVATED PROTEIN KINASE 3 (MPK3)*	AT3G45640	2.79	3.08
	*PROPEP 1*	AT5G64900	3.24	4.03
	*PROPEP 4*	AT5G09980	3.31	8.84
Cell wall associated defense	*CELLULOSE SYNTHASE A4 (CESA4)*	AT5G44030	2.43	2.60
	*CELLULOSE SYNTHASE 8 (CESA8)*	AT4G18780	2.21	2.42
Plant Defensins	*PLANT DEFENSIN 1*.*4 (PDF1*.*4)*	AT1G19610	9.66	2.92
	*PLANT DEFENSIN 2*.*2 (PDF2*.*2)*	AT2G02100	4.00	4.84
Jasmonate associated genes	*MYC 2*	AT1G32640	2.34	2.39
	*JASMONATE-ZIM-DOMAIN PROTEIN 1 (JAZ1)*	AT1G19180	2.16	2.22
	*JASMONATE-ZIM-DOMAIN PROTEIN 5 (JAZ5)*	AT1G17380	2.96	3.51
	*JASMONATE-ZIM-DOMAIN PROTEIN 7 (JAZ7)*	AT2G34600	3.20	3.82
	*JASMONATE-ZIM-DOMAIN PROTEIN 8 (JAZ8)*	AT1G30135	2.39	3.17
	*JASMONATE-ZIM-DOMAIN PROTEIN 10 (JAZ10)*	AT5G13220	4.44	5.60
	*DONGLE (DGL)*	AT1G05800	4.42	4.68
	*ALLENE OXIDE SYNTHASE (AOS)*	AT5G42650	2.25	2.38
	*ALLENE OXIDE CYCLASE 3 (AOC3)*	AT3G25780	2.43	2.96
	*LIPOXYGENASE 4 (LOX4)*	AT1G72520	2.82	3.39
	*SULFOTRANSFERASE 2A (ST2A)*	AT5G07010	2.99	2.53
	*CYTOCHROME P450*, *FAMILY 94*, *SUBFAMILY B3 (CYP94B3)*	AT3G48520	5.29	6.88
	*VEGETATIVE STORAGE PROTEIN 1 (VSP1)*	AT5G24780	5.01	7.22
	*VEGETATIVE STORAGE PROTEIN 2 (VSP2)*	AT5G24770	3.61	3.25

The reduced expression of JA signaling genes such as *MYC2* and the *JAZ* genes in *med18* and *med20* suggests that the MED18 and MED20 subunits are required for maintaining a functional JA signaling pathway. Interestingly, well-studied JA/ET-associated defense marker genes such as *PLANT DEFENSIN1*.*2*, (*PDF1*.*2*), the *BASIC CHITINASE* (*CHI-B*) and *HEVEIN-LIKE* (*HEL*) *PR* genes were not found to be differentially regulated in the RNAseq experiment. Overall the RNAseq and RT-qPCR results suggest that the *MYC2* regulated branch of JA signaling is affected in *med18* and *med20* [45, 46] which may potentially explain the strong *F*. *oxysporum* resistance displayed by these mutants.

The GO analysis of genes expressed higher in the *med18* and *med20* roots versus WT roots identified GO terms associated with transcription and RNA metabolism as well as the GO terms associated with ion and multi-drug transport. One of the transporters significantly induced was *PDR12*, a pathogen and defense hormone inducible gene encoding an ABC transporter [30]. As would be expected with alterations in the Mediator complex, several transcription factors were affected in the *med18* and *med20* mutants, with WRKYs, MYBs, NACs and MADS box TFs being identified as differentially expressed. GO terms associated with oxidative stress, hydrogen peroxide and ROS were also identified in the *med20* induced gene list but not in *med18*. Overall, the GO enrichment analysis identified response to fungi, chitin and JA-associated gene expression as being higher in the WT, whereas the *med18* and *med20* mutants showed transcription, and transporter associated GO terms being enriched with additional ROS associated GO terms identified in the *med20* mutant.

### *MED18* and *MED20* negatively regulate SA-associated defence genes

**Fig 6:**
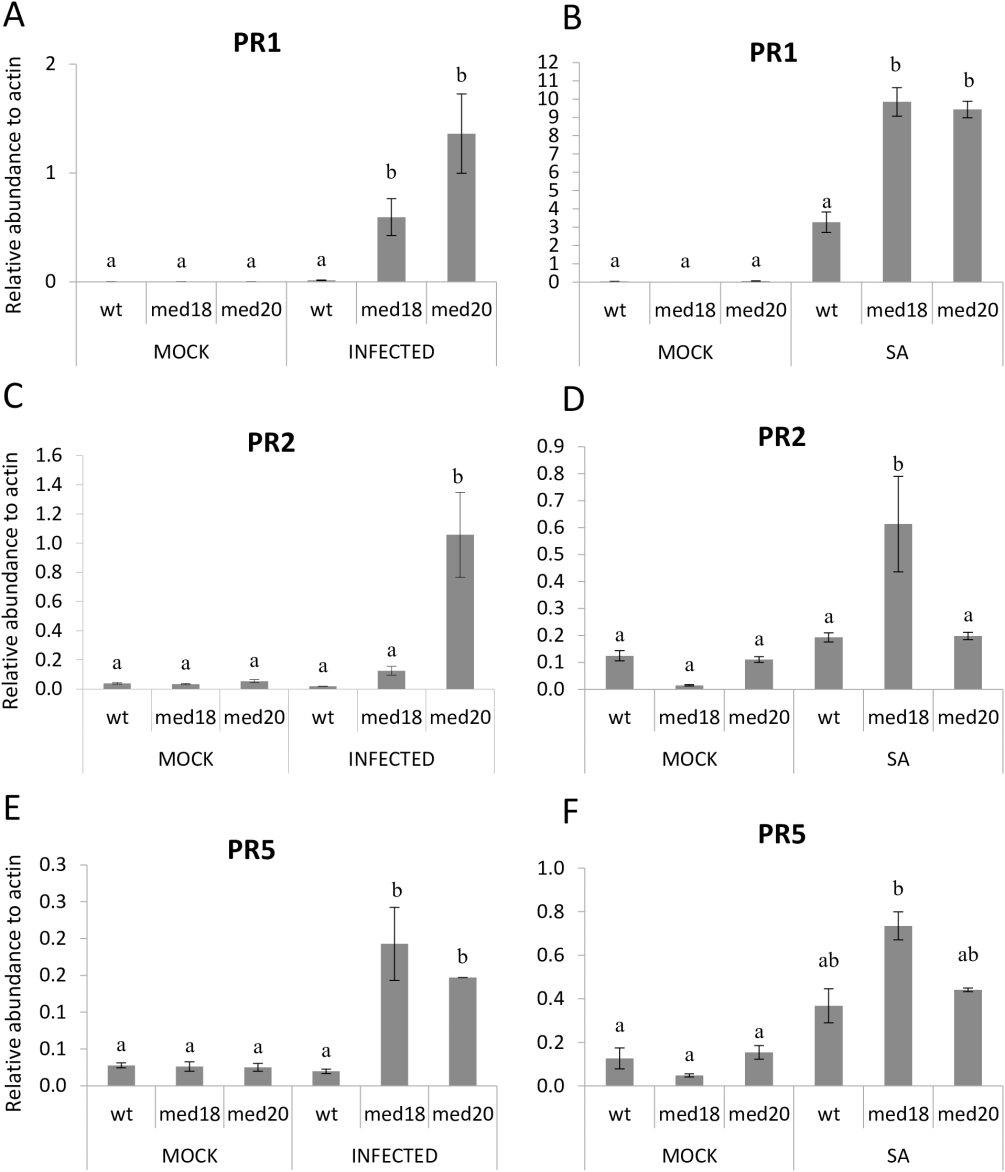
Real time qRT-PCR data of SA-associated genes in WT, *med18* and *med20* roots and leaves. (A, C, E) *PR1*, *PR2* and *PR5* expression in roots of WT, *med18* and *med20* with and without *F*. *oxysporum* infection. (B, D, F) *PR1*, *PR2* and *PR5* expression in leaves of WT, *med18* and *med20* in response to 24 hours of mock or SA treatment. Results were obtained from three independent biological replicates of twenty plants per replicate. (a & b) indicates *p*-value < 0.05 using a one-way ANOVA, least significant difference test. Error bars represent standard deviation of the biological replicates.

As the JA and SA signaling pathways often antagonize each other, we examined whether SA- associated defense genes were activated in *med18* and *med20* plants. We examined the expression of SA- associated *PR* genes, *PR1*, *PR2* and *PR5* with RT-qPCR and found that *PR1* and the thaumatin-like *PR5* gene was significantly induced in *med18* and *med20* roots relative to the WT after *F*. *oxysporum* infection. *PR2* was also found to be induced in both *med18* and *med20*, but the induction was not statistically significant in *med18* (Fig 6). We examined these same genes in leaf tissue after treatment with SA to see if the *med* mutants had higher SA regulated gene expression in other tissues. These experiments showed that *PR1* expression was approximately three fold higher in both mutants after SA treatment. *PR5* was not significantly induced in either mutant, while *PR2* was expressed higher in *med18* leaves only (Fig 6). Therefore different patterns of *PR* gene induction are seen in the *med18* and *med20* leaves after SA induction as opposed to in roots after *F*. *oxysporum* infection. This is not surprising as infection with *F*. *oxysporum* would induce a response in gene expression based on a variety of elicitors and effectors as opposed to a single hormone treatment with SA. Overall we conclude that *PR1* and *PR5* are induced higher in *med18* and *med20* in response to *F*. *oxysporum* infection at early time points and may play a role in restricting pathogen colonization in the roots.

**Fig 7:**
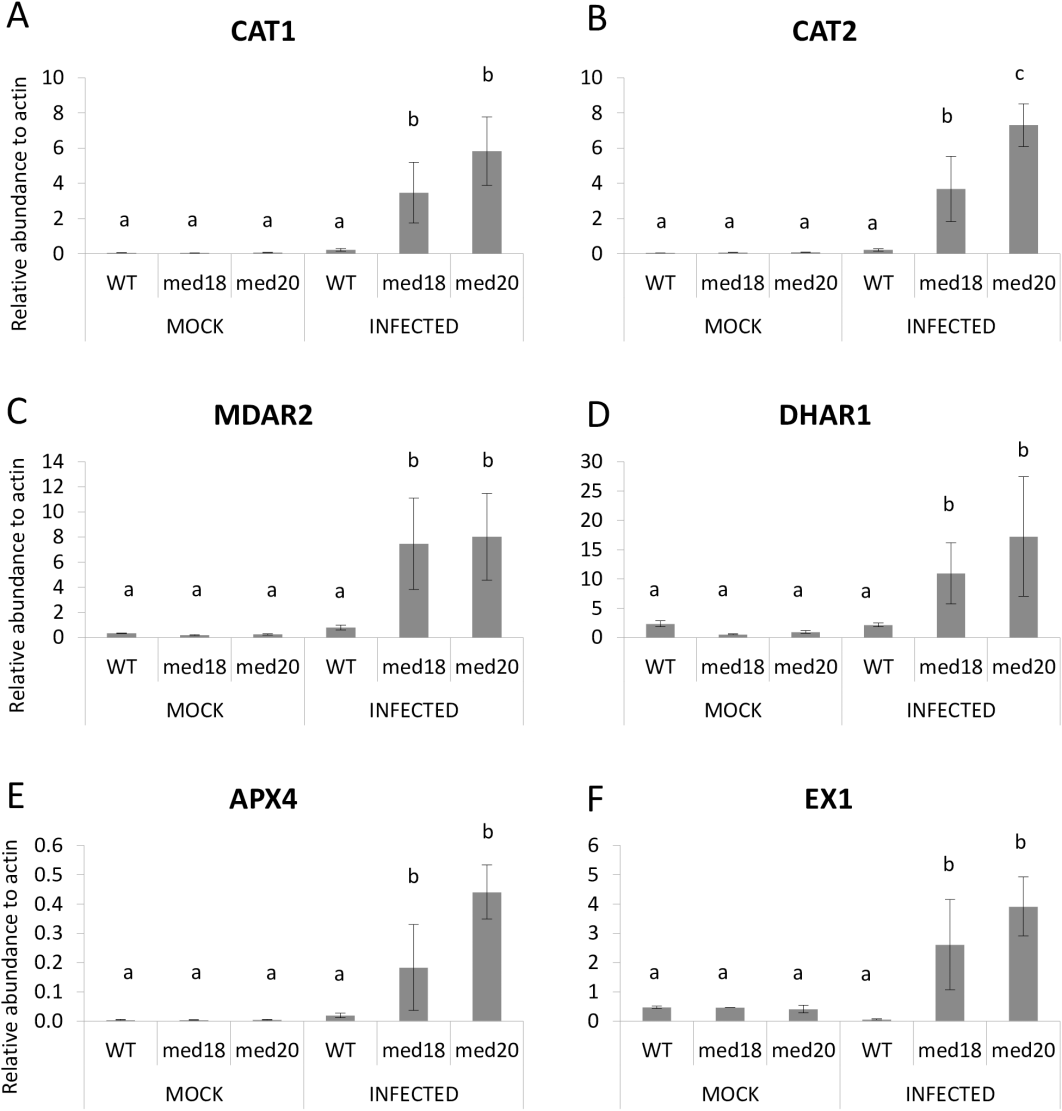
ROS associated genes that are expressed higher in *med18* and *med20* roots in response to *F*. *oxysporum* infection. (A-F) The expression of *CAT1*, *CAT2*, *MDAR2*, *DHAR1*, *APX4* and *EX1* was significantly higher in *med18* and *med20* roots than the WT after 24 hours *F*. *oxysporum* infection. Results were obtained from three independent biological replicates of twenty plants per replicate. (a, b & c) indicates *p*-value < 0.05 using a one-way ANOVA, least significant difference test. Error bars represent standard deviation of the biological replicates.

Activation of plant defenses by biotrophic fungal pathogens is often associated with increased SA-defenses, ROS production and the hypersensitive response in plants [47]. Response to ROS was also a significantly enriched GO term enriched in *med20* versus the WT (S3 Table). To investigate and compare the involvement of ROS in the hemi-biotrophic infection of *F*. *oxysporum*, we conducted RT-qPCR expression analyses of genes known to have a role in ROS generation or metabolism. Genes found to be induced higher in *med18* and *med20* were the ROS scavenging *CATALASE* genes *CAT1* and *CAT2*, as well as the oxidoreductases *MONODEHYDROASCORBATE REDUCTASE2* (*MDAR2*), *DEHYDROASCORBATE REDUCTASE1* (*DHAR1*) and *ASCORBATE PEROXIDASE4* (*APX4*) (Fig 7; [48]). Also expressed higher was *EXECUTER1* which plays an important role in singlet oxygen stress [49, 50].

**Fig 8:**
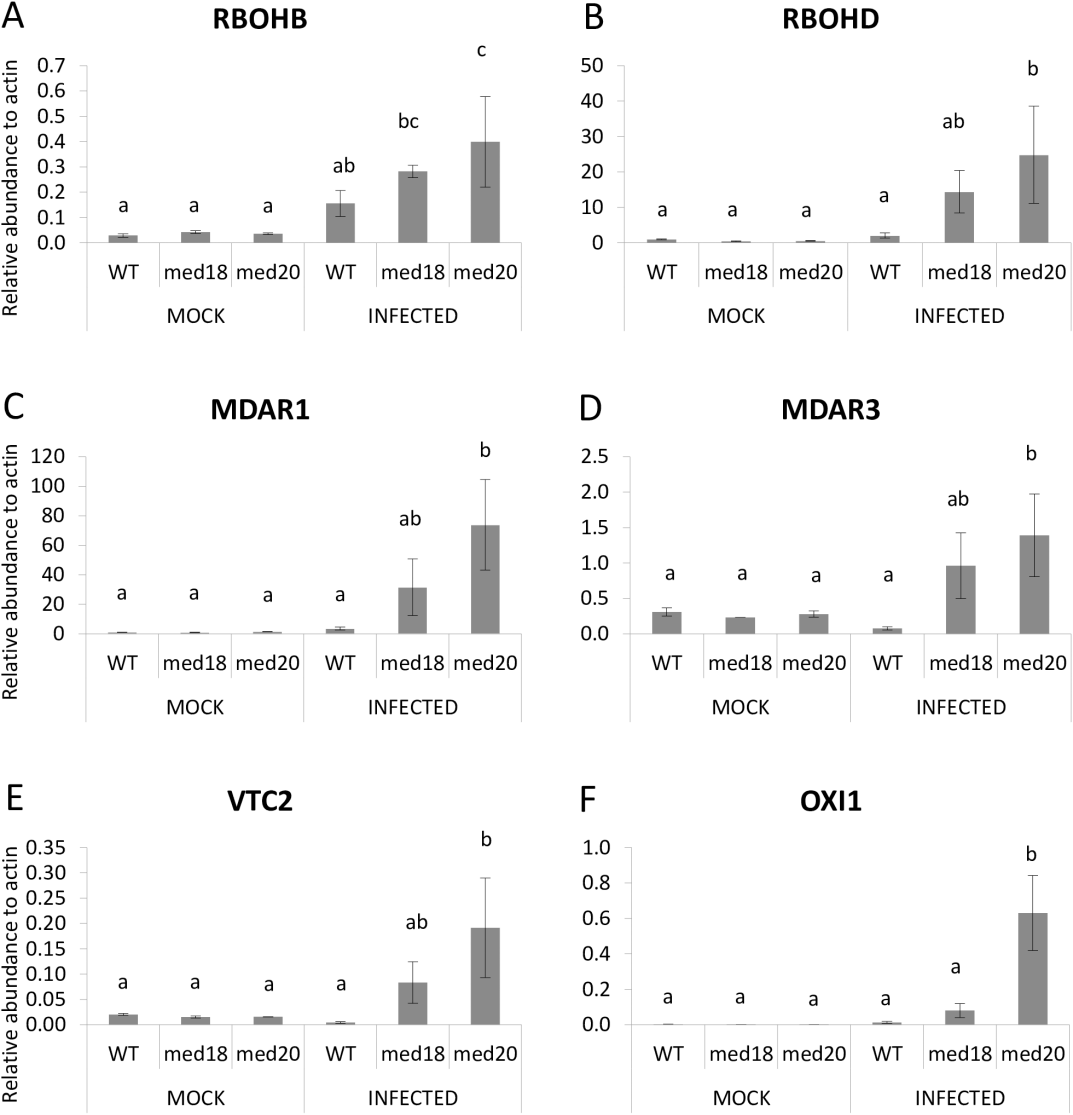
ROS associated genes that are expressed higher in *med20* roots in response to *F*. *oxysporum* infection. (A-F) The expression of *RBOHD*, *RBOHF*, *MDAR1*, *MDAR3*, *VTC2* and *OXI1* was significantly higher in *med20* roots after 24 hours *F*. *oxysporum* infection. Results were obtained from three independent biological replicates of twenty plants per replicate. (a, b & c) indicates *p*-value < 0.05 using a one-way ANOVA, least significant difference test. Error bars represent standard deviation of the biological replicates.

In addition, several genes were found to be significantly up-regulated only in *med20*, including *RESPIRATORY BURST OXIDASE HOHMOLOGS*, *RBOHB* and *RBOHD*, *MONODEHYDROASCORBATE REDUCTASE1*, *MONODEHYDROASCORBATE REDUCTASE3*, *VITAMIN C DEFECTIVE2* and *OXIDATIVE SIGNAL INDUCIBLE1* (Fig 8). The up-regulation of *EX1* and ROS quenching genes such as the *CATALASE* and ascorbate reductase genes in both mutants suggest the possibility for increased ROS production in *med18* and *med20* roots in response to *F*. *oxysporum*.

## Discussion

Since the discovery of the Mediator complex in Arabidopsis [51], individual subunits of the complex have been shown to be involved in a wide range of biological processes in plants [25]. Through screening of twelve T-DNA insertion lines in Arabidopsis Mediator genes, we previously identified the *MED8* and *MED25* subunits to be required for susceptibility to *F*. *oxysporum* [26]. While the *F*. *oxysporum* resistance phenotype of the *med25* mutant was linked to a defect in JA signaling, the *med8* mutant demonstrated wild type levels of JA/ET defense marker gene expression [26]. To further investigate the role of Mediator subunits in *F*. *oxysporum* resistance, we inoculated mutants of the head domain subunits *MED18* and *MED20*, which are predicted to be located adjacent to *med8* in the Arabidopsis Mediator complex. Interestingly, the *med18* and *med20* mutants were found to be phenotypically similar to each other with almost complete resistance to *F*. *oxysporum*. The *med8*, *med18*, *med20*, *med25 mutants* and *med8 med25* double mutant all display a delay in flowering time and enhanced resistance to *F*. *oxysporum*. It was previously hypothesized that a delay in flowering time might lead to enhanced resistance to *F*. *oxysporum*, as several mutants with delayed flowering time (e.g. *myc2*, *arf2*, *fve-3*), as well as Arabidopsis accessions with delayed flowering also have enhanced resistance [40]. However the link between flowering time and *F*. *oxysporum* resistance could be broken by removing the flowering repressor *FLOWERING LOCUS C* (*FLC*) in the *fve-3* mutant or through vernalization in some natural accessions [40]. We found that the flowering time and *F*. *oxysporum* phenotypes are spatially separate in *med18* using grafting. The flowering time phenotype was found to be regulated by the leaves as expected given that this is where the expression of *FLOWERING LOCUS T* (*FT*) is triggered [52], whereas *F*. *oxysporum* resistance occurs in the roots, resulting in failed root colonization in *med18* roots.

Through RNAseq analysis we observed a significant reduction in JA-associated genes in *med18* and *med20*, relative to the WT. This analysis identified genes affecting JA accumulation such as *AOS*, *AOC3*, *LOX4*, *DONGLE*, and a JA-Ile-hydroxylase as well as JA-signaling genes such as *MYC2* and the *JAZ* genes. These results suggest that MED18 and MED20 are involved in regulating JA signaling in *Arabidopsis*. In accordance with previous findings on antagonistic interactions between JA- and SA-associated defense pathways, the expression of *PR1* and *PR5* was higher in *med18* and *med20* in response to *F*. *oxysporum* treatment. We also found up-regulation of singlet oxygen stress responsive genes, *EX1*, in *med18* and *med20* and *OXI1* in *med20* only, as well as up-regulation of ROS scavenging genes such as *CAT1* and *CAT2* and the ascorbate reductases in both mutants. *OXI1* and *EX1* have been recently shown to regulate singlet oxygen mediated cell death through independent pathways [50, 53]. Jasmonate has also been shown to play a role in singlet oxygen responses, which has been revealed through recent investigations with the *flu* and *ch1* mutants (reviewed by [54]). It is possible that both JA signaling and singlet oxygen stress signaling is channelled through the Mediator complex via MED18 and MED20 or alternatively, mis-regulation of the JA pathway in *med18* and *med20* leads to defects in ROS production and tolerance. With both ROS producing and ROS scavenging genes being upregulated at the same timepoint, further work examining additional timepoints is needed to investigate the amplitude and types of ROS that are produced and whether altered ROS levels in *med18* or *med20* impact on *F*. *oxysporum* directly or instead play a role in defense signaling.

In addition to being more resistant to *F*. *oxysporum*, mutants of *MED8*, and *MED18* have been shown to be susceptible to necrotrophic leaf pathogens such as *Alternaria brassicicola* and *Botrytis cinerea* [26, 39, 55]. Recently it was shown that MED18 is recruited by the histone acetyltransferase, HOOKLESS1, to the *WRKY33* promoter and thereby increases *WRKY33* expression [55]. WRKY33 has an important role in JA/SA crosstalk as well as redox homeostasis, [56]. Loss of WRKY33 results in activation of SA defense responses and down-regulation of JA-associated responses and therefore the JA/SA crosstalk we observed here could be associated with MED18’s recruitment to the *WRKY33* promoter. *WRKY33* was not differentially regulated in the RNAseq experiment at 24hours, but future work should examine whether changes in expression of this gene and other TFs with a role in cross talk are seen at earlier or later timepoints. MED18 has also been found to interact with TFs such as YIN YANG1, ABA INSENSITIVE4 and SUPPRESSOR OF FRIGIDA4 [39] and it would be important to test whether these TFs also affect *F*. *oxysporum* resistance. Alternatively, as mutations in *med18* and *med20* in the yeast Mediator complex affect the stabilization of RNA Pol II and TFIIB interactions [29] it is possible that disruption to this subdomain leads to a change in the binding surface which might subsequently affect these phenotypes through indirect mechanisms such as reduced RNA Pol II occupancy and altered histone modifications as has been demonstrated [39, 55].

Recently, the isolation of several mutants in an RNA binding KH domain protein termed *SHINY1*/*ENHANCED STRESS RESPONSES1* (*SHINY/ESR1*) was identified through a forward genetic screen [57]. The *esr1* mutants were found to have increased resistance to *F*. *oxysporum* as well as differential regulation in some but not all aspects of the JA pathway [57]. Previously SHINY1/ESR1 was shown to interact with FIERY2*/*RNA POLYMERASE II CARBOXYL TERMINAL DOMAIN PHOSPHATASE-LIKE 1 (FRY2/CPL1), a protein that de-phosphorylates the C terminal domain (CTD) of RNA Pol II [58, 59]. CPL1 has been shown to be essential for accurate miRNA processing and controls mRNA splicing and mRNA decay [60–62]. It has previously been shown that mutations in *MED18* and *MED20* also affect miRNA levels and a mutation in RNA Pol II leads to similar developmental phenotypes as *med18* and *med20* suggesting a connection between mutations in RNA binding proteins [57], the Mediator head domain and mutations in RNA Pol II itself [31]. This suggests that disruptions in this region of the RNA pol II holoenzyme result in similar developmental and biotic phenotypes in *Arabidopsis*.

While we detected interaction between MED18 and MED20 in reciprocal Yeast-2-Hybrid experiments, we were unable to identify a positive interaction with MED8. This was surprising as MED18 and MED20 form part of the movable jaw that connects through the C-terminal portion of MED8 in the yeast Mediator complex. However it has been shown that correct folding of yeast MED8, MED18 and MED20 as a trimer requires all three proteins [63], and therefore it is possible that all three proteins are required for proper interaction of the *Arabidopsis* proteins as well. Recent structural modelling of the yeast Mediator complex show MED18 and MED20 form a separate interface with RNA pol II as compared to MED8 [29]. MED18 and MED20 form an interface with RPB3 and RPB11 of RNA pol II, and the TFIIB B–ribbon domain, whereas MED8 forms interactions with the RPB4-RBP7 stalk of RNA pol II [29]. The Mediator complex has shown remarkable structural conservation across yeast and metazoan complexes [64, 65], and it is hypothesized that a similar structural organization occurs in the *Arabidopsis* complex. As *in vivo* protein pull down experiments with *Arabidopsis* Mediator subunits results in the entire complex being isolated [51], more detailed structural biology approaches are required to identify where MED18 and MED20 sit in the *Arabidopsis* Mediator complex and how disruption of either MED18 or MED20 subunits affects their interaction with the *Arabidopsis* RNA Pol II holoenzyme.

Overall we propose that MED18 and MED20 form a sub-domain within the *Arabidopsis* Mediator complex that regulates flowering time and pathogen defense. A reduction in JA signaling and enhanced SA- and ROS-associated gene expression was observed in both mutants which might contribute to the restriction of *F*. *oxysporum* growth during root infection. Further work should reveal how MED18 and MED20 dock in the *Arabidopsis* Mediator complex and provide further insights into the mechanistic process for producing the strong *F*. *oxysporum* resistance phenotype observed.

## Supporting information

S1 Fig Flowering phenotypes of grafted *med18* and WT. Photographs were taken two weeks after infection with *F*. *oxysporum*.
(PPTX)

S2 Fig RT-qPCR data for JA-associated genes in WT, *med18* and *med20* roots with and without *F. oxysporum* infection. Results were obtained from three independent biological replicates. ANOVA, LSD significant difference test (a, b, & c) indicates *p*-value < 0.05; error bars represent standard error of the biological replicates.
(PPTX)

S1 Table Primers used in this study for RT-qPCR and cloning of MEDIATOR genes. (XLSX)

S2 Table Differentially expressed gene list comparing WT with *med18* and *med20* from the RNAseq analysis. (XLSX)

S3 Table GO term enrichment analysis of the RNAseq experiment. (XLSX)
