# Supplementary figures and images for: MEDIATOR18 and MEDIATOR20 confer susceptibility to Fusarium oxysporum in Arabidopsis thaliana
Source: PLoS One. 2017 Apr 25;12(4):e0176022. doi: 10.1371/journal.pone.0176022 (PMC5404846; doi:10.1371/journal.pone.0176022)

## Slide 1
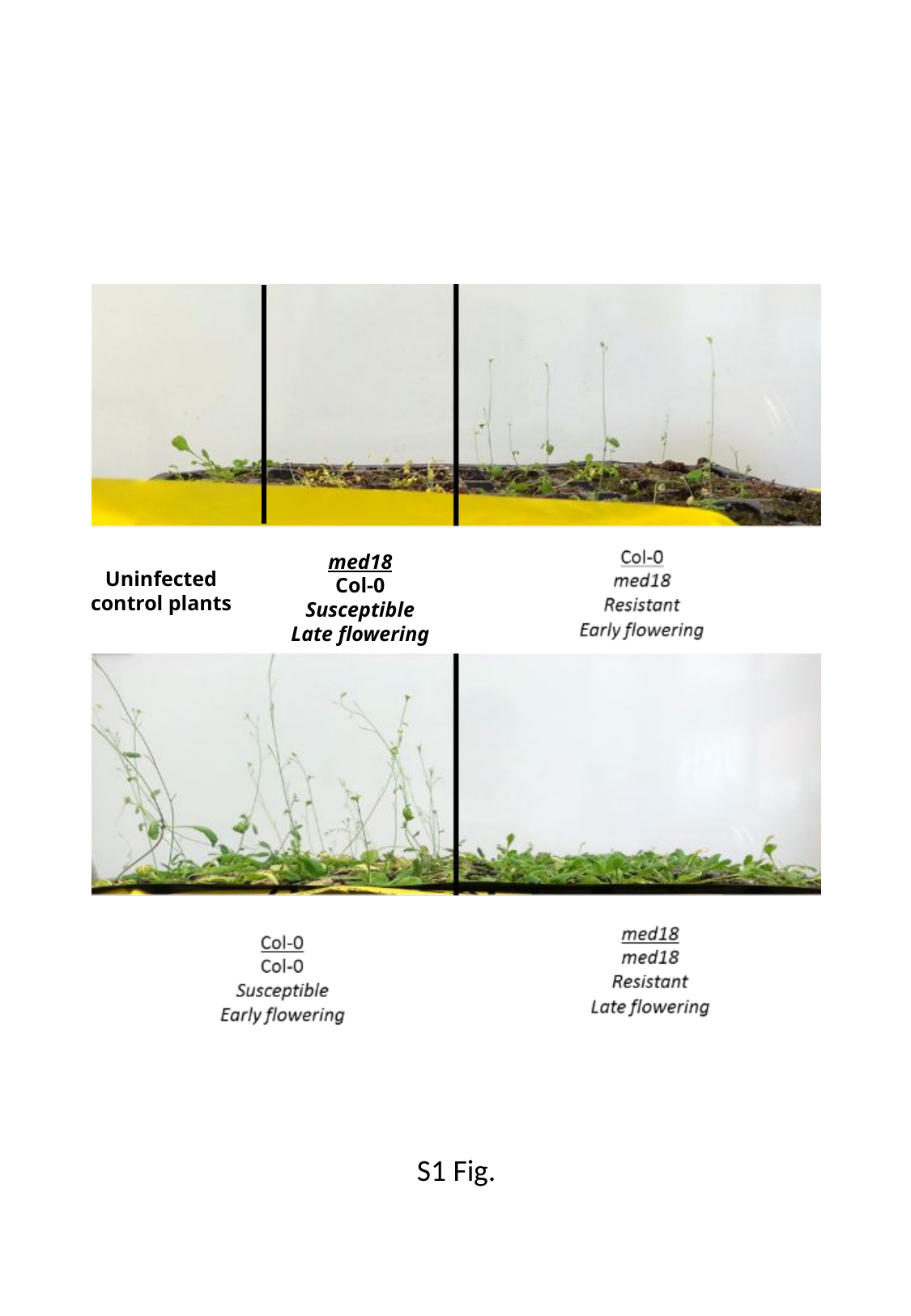

med18
Col-0
Susceptible
Late flowering
Uninfected
control plants
S1 Fig.

Supplement: S1 Fig — Photographs were taken two weeks after infection with F. oxysporum. (PPTX) [file pone.0176022.s001.pptx]
