# Supplementary material for: MEDIATOR18 and MEDIATOR20 confer susceptibility to Fusarium oxysporum in Arabidopsis thaliana
Source: PLoS One. 2017 Apr 25;12(4):e0176022. doi: 10.1371/journal.pone.0176022 (PMC5404846; doi:10.1371/journal.pone.0176022)
Supplement: S2 Fig — Results were obtained from three independent biological replicates. ANOVA, LSD significant difference test (a, b, & c) indicates p-value < 0.05; error bars represent standard error of the biological replicates. (PPTX) [file pone.0176022.s002.pptx]

## Slide 1
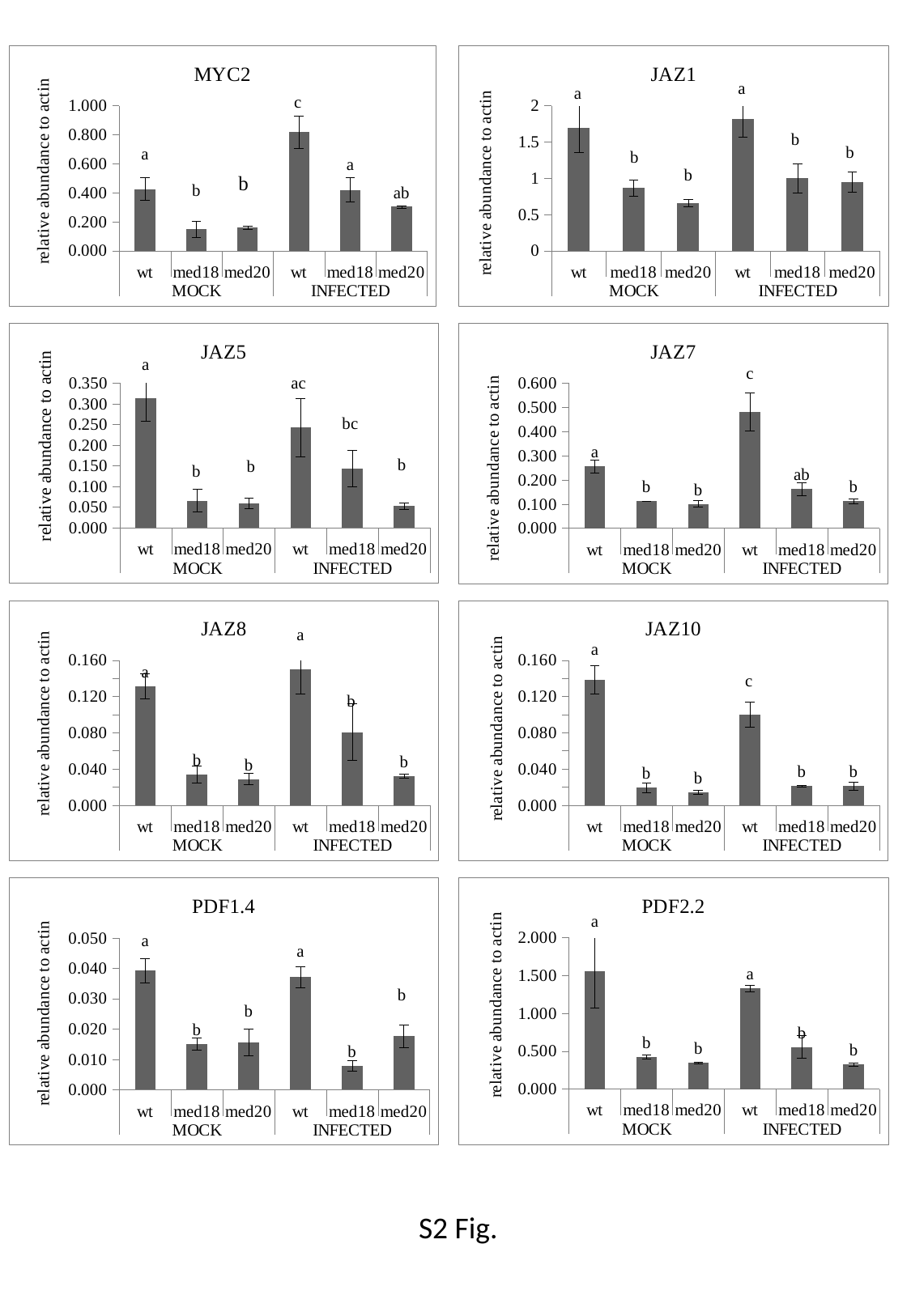

### Chart: MYC2
| Category | myc2 |
|---|---|
| wt | 0.425657013710325 |
| med18 | 0.146648689254268 |
| med20 | 0.160105001358126 |
| wt | 0.820620772048808 |
| med18 | 0.419711234588881 |
| med20 | 0.303387837361616 |
### Chart: JAZ1
| Category | JAZ1 |
|---|---|
| wt | 1.69845648429835 |
| med18 | 0.867176635280067 |
| med20 | 0.659834309977977 |
| wt | 1.823152099572802 |
| med18 | 1.000028144168053 |
| med20 | 0.951656560612266 |
### Chart: JAZ5
| Category | JAZ5 |
|---|---|
| wt | 0.312757609566793 |
| med18 | 0.066038804893418 |
| med20 | 0.0597153477459487 |
| wt | 0.242170128166845 |
| med18 | 0.1442288230003 |
| med20 | 0.0534530868473058 |
### Chart: JAZ7
| Category | JAZ7 |
|---|---|
| wt | 0.255736915619913 |
| med18 | 0.11338551935462 |
| med20 | 0.10104088088841 |
| wt | 0.48322877985497 |
| med18 | 0.162341241973466 |
| med20 | 0.112764065300371 |
### Chart: JAZ8
| Category | JAZ8 |
|---|---|
| wt | 0.131510507440773 |
| med18 | 0.03375032850718 |
| med20 | 0.0288991212026559 |
| wt | 0.149630690216411 |
| med18 | 0.0806652824933332 |
| med20 | 0.0322553539290578 |
### Chart: JAZ10
| Category | JAZ10 |
|---|---|
| wt | 0.138320013499653 |
| med18 | 0.0193477626271832 |
| med20 | 0.0142994543402454 |
| wt | 0.100236087356275 |
| med18 | 0.0210327845734734 |
| med20 | 0.0211868745629225 |
### Chart:
| Category | PDF1.4 |
|---|---|
| wt | 0.0392648355570636 |
| med18 | 0.0150619067639137 |
| med20 | 0.0156657223580377 |
| wt | 0.0370776173323972 |
| med18 | 0.00789070205806663 |
| med20 | 0.0177342227325595 |
### Chart: PDF2.2
| Category | PDF2.2 |
|---|---|
| wt | 1.560193654701813 |
| med18 | 0.425894771014611 |
| med20 | 0.34578665796576 |
| wt | 1.332156601139854 |
| med18 | 0.555768376779127 |
| med20 | 0.326891609929858 |S2 Fig.
